# Correspondence

**Published:** 1910-04

**Authors:** 


attendance, it was decided the best interests of the State Association would be served by postponing the annual session until a later date. September provides a number of attractions and is sufficiently remote to warrant a second visit to the metropolis of those who attend the Rose Festival. Of these attractions, probably no other will provide better entertainment than the Country Club, and the Officers of the State Medical Association congratulate themselves on the dates selected. President Pierce intends to make the approaching meeting memorable. Negotiations are pending with noted Eastern men who are expecting to attend. No effort is being spared to secure papers of unusual merit, in which Dr. Pierce is being backed by the officers and members of the Association to the limit. If you are a member of the Oregon Association, plan to come; if you are a member of the Association of any neighboring state, come; if you are not a member of any of these, come anyway. You will be compensated for your time and trouble and you will be thrice welcome.

W. H.

---

### ARE THE ELECTIONS IN THE A. M. A. AND ITS CONSTITUENT ASSOCIATIONS FAIR OR LEGAL?

*Editor Atlanta Journal-Record of Medicine*:

As is well known, I have for some time been endeavoring to institute a reform movement in the politics of the A. M. A. and its constituent bodies. In furtherance of this movement I herewith submit to various journals for publication certain facts which, it seems to me, should prove to any reasonable or fair-minded man the logic of my contentions:

1st. The voting unit of the A. M. A. is the legally qualified member of the district or county society. If this voting unit is not a voting member of the A. M. A. he is not legally qualified to vote for any officer who functionates as an elector in the A. A. M. A. Note, please, the following:

*a.* The voting unit in the district or county society votes for a councillor or councillors.

*b.* The council votes for State delegates.

*c.* The States delegates vote for delegates to the A. M. A.

*d.* The delegates to the A. M. A. elect its officers and through these officers transact its business.

*The voting units are largely non-members of the A. M. A. and not legally qualified to serve as electors in that body.*

According to Dr. W. B. Dorsett, of St. Louis, Mo., 295 out of 747 members of the St. Louis Society are non-members of the A. M. A. and therefore not qualified to vote for any officer who, or policy which, is directly or indirectly concerned in the official or business management of the A. M. A. *And yet they do vote, the members of the A. M. A. paying a premium for political rights which are given to non-members free of charge so far as the elective franchise in the A. M. A. is concerned.* What is true of St. Louis is true of Chicago and every other large city. In the Chicago Medical Society there are 503 non-members of the A. M. A.—these are all illegal voting units of the A. M. A. *This throws the balance of power into the hands of the city members of the A. M. A. with a vengeance, and it is a power which can readily be abused.*

According to the A. M. A. directory there are 73,478 members of State societies in the United States. Of these, only about 35,000 are members of the A. M. A. More than fifty per cent., therefore, of the total votes cast in A. M. A. politics are illegal and absolutely unfair. In Illinois there are 5,246 physicians who are members of the State society. Only 3,310 of these are members of the A. M. A. Nearly 2,000 men, therefore, vote or are permitted to vote in A. M. A. politics in Illinois alone, who have no legal or moral right to vote. Nearly 50 per cent. of the voters in the New York State society are non-members of the A. M. A., hence vote illegally. When one considers the enormous financial and professional interests of the A. M. A., these figures are startling.

2nd. Affiliated bodies.

I doubt if history can show a more ludicrous political farce then the affiliated special societies in the A. M. A. There are ten in Chicago, each represented by a councillor. One may belong to as many as he likes—to all, if the men in control of these organizations see fit to use them for political purposes. As each member is supposed to be a member and voting unit in his local society, he has as many votes in A. M. A. politics and in the constituent bodies— local and State societies—as he has memberships in the affiliated bodies. Thus, in Chicago, if he belongs to ten, he has ten votes for councillor in the affiliated societies; one for councillor of his branch society and one for councillor at large. *Thus he has twelve votes to the ordinary members two!* Should he himself chance to be first, a councillor, second, a State delegate, third, an A. M. A delegate, *he has fifteen votes in all to the ordinary members two!* Should he chance to be an officer of the A. M. A. *this means fifteen votes for himself!*

The local trustee of the A. M. A. in Chicago has been accredited with membership in five affiliated bodies. As he is a councillor, and State delegate, *his total vote—for himself and coterie of supporters is therefore,* 9!

Can the illegal vote of the affiliated bodies be controlled and used for partisan political purposes? Very easily—in fact, it is so used. *Of the eighteen councillors who voted against the A. M. A reform resolutions recently adopted by the Chicago Medical Society, nine were from the affiliated bodies*!

Comment would be superfluous. I would merely remark that the situation should be quite comforting to those who are inspired by political greed and desire for monopoly, but somewhat disquieting to the country members of the A. M. A., and especially to the members of the various State societies who are not members of affiliated bodies or even of the A. M. A., and who must perforce be content with a single vote— a pitiful crumb from the political table. Their illegal vote in the State society for A. M. A. representation should not console the latter class for the impositions put upon them in local and State politics.

As matters now stand, neither the A. M. A. nor the State societies are autonomous. *To pile Pelion upon Ossa, matters are so arranged that men in the affiliated bodies who are not even members of the Chicago Medical Society vote for councillors from the affiliated bodies*!

Preposterous, but true. The affiliated bodies in Chicago—some of them, at least—demand that an applicant for membership shall be a member of the Chicago Medical Society. *If, however, he drops out of the Chicago Medical Society, his elective franchise is not taken away from him!*

The spectacle of officers of the A. M. A. serving as, first, councillors, second, State delegates, third, national delegates, is decidedly un-American and *very disquieting to one who has pride of citizenship and believes in equal rights and fair play in our* medical societies.

Remedies:

1st. Exclusion of the affiliated bodies from illegal and unjust political privileges, their members having only the same rights in the local societies as other members.

2nd. Membership ballot in the State societies.

3rd. Membership ballot in the A. M. A.

*The only alternative for the ballot would be to compel all members of the State societies to join the A. M. A., for which alternative, my brethren, you would not stand for a moment.* The voting should be done by the members in attendance at the meeting. This would put a premium on attendance and increase both attendance and enthusiasm. Nominations should be made on the first day and voting done on the succeeding days of the meeting. A certain number of nominations by petition would aid in guarding membership rights and democratizing our politics. The place of registration could be made the polling place. The A. M. A., the State societies and the local societies should be made autonomous if we are to avoid the rocks and shoals of political unfairness, absurdity and corruption—above all, if we would avoid despotism. The ballot having been obtained, one thing more is necessary to secure autonomy for the constituent societies, viz.: *All officers of the A. M. A. must be prohibited from holding multiple offices and more especially from serving as councillors and State or national delegates.*

Let the rank and file carefully consider the foregoing. Not to-day, nor perhaps to-morrow, but some time in the near future, the members of the local, State and national associations will recall what is here written and wonder why they did not sooner awaken from their lethargy and spiritless submission to almost incredible political conditions which have robbed them of their rights of citizenship as well as of the society membership rights for which they yearly pay their good money.

I have no axe to grind, my brethren, and once the necessary reforms have been established I shall return to more congenial pursuits than medico-political battles. It matters naught to me personally whether the members of the State societies go on and on forever, blindly following the lead of professional medical politicians, but it matters much to the rank and file whether the local and State organizations continue to be merely kitchens for A. M. A. politics. If the members of the State societies are content to allow the State organizations to continue to be merely adjourned meetings of A. M. A. officials, or at least of the large city societies, I presume that I ought to be reconciled to the situation. And yet, as a matter of justice to the rank and file, I am not.

G. FRANK LYDSTON, M. D.
Chicago.

